# E-cigarette use and knowledge of its effect on oral health among health sciences students in Trinidad and Tobago

**DOI:** 10.3389/froh.2025.1547246

**Published:** 2025-06-30

**Authors:** Cherisse Z. Beard, Anil J. Ragbir, Tia L. Rice, Makiba M. Thomas, Rahul S. Naidu

**Affiliations:** The School of Dentistry, Faculty of Medical Sciences, The University of the West Indies, St. Augustine, Trinidad and Tobago

**Keywords:** e-cigarette, vaping, smoking, tobacco, nicotine, oral health, Caribbean

## Abstract

**Background:**

Electronic cigarette (e-cigarette) use has become more common among younger age groups around the world. Health concerns, including oral health effects have been reported.

**Objective:**

This research aimed to assess prevalence of e-cigarette use and knowledge of oral health effects among young university students in Trinidad and Tobago and implications for oral health promotion.

**Methods:**

A sample of 193 students, >18 years old, consisting of health sciences students from the Faculty of Medical Sciences, the University of the West Indies. Participants completed an online self-administered questionnaire including demographics, e-cigarette use habits and effect on oral health.

**Results:**

The majority were female participation (75.1%). Age range of participants was 19 to 25-years-old, mean age 22 years old. 15% of participants currently used e-cigarettes. Most participants believed that e-cigarette use affects health (97.9%), is possibly addictive (91.2%), delivers nicotine (87%) and contains chemicals (87.6%). 51.3% believed that e-cigarette use, related to tooth decay and 58.5% gum disease. 44% of participants were not familiar with research on e-cigarette use and its effects on oral health. Most participants thought the information on e-cigarettes was important (78.3%) and (71.5%) would speak to a dentist about its effects on oral health. 76% of participants indicated willingness to quit e-cigarette use if adverse oral health effects were understood.

**Conclusion:**

Among this sample of health sciences students, e-cigarette use was low. There was limited knowledge of its effects on oral health. Adverse effects of e-cigarette use on oral health should be included in oral health promotion initiatives.

## Introduction

Electronic cigarette (e-cigarette) use has become a popular trend and marketed as a safer alternative for nicotine delivery compared to traditional tobacco smoking since its inception almost twenty years ago (1). These products are also known as vapes, vape pens, e-cigars, electronic hookahs and pod systems, can be categorized as Electronic Nicotine Delivery Systems and Electronic Non-Nicotine Delivery Systems (ENDS and ENNDS) depending on if they contain nicotine or not along with other components such as chemical additives and flavourings which, when heated produce aerosols that are inhaled (2, 3). The enticing flavours, sleek design and the perception that it is less harmful than conventional tobacco products have attracted much of the younger population worldwide (2–4). However, there is emerging evidence that highlights the potential negative health effects these e-cigarettes pose, including oral health effects (3). Initially e-cigarettes have been used to help tobacco smokers gradually quit smoking as they were marketed as a healthier alternative and for this reason they gained popularity (5–7). Exposure to components of e-cigarettes may have health effects (1, 8). Research has shown a link between e-cigarette use and e-cigarette or vaping product use-related-associated lung injury (EVALI), a form of acute lung injury (9, 10).

Research has found negative effects on oral health caused by e-cigarettes. These included increased risk of periodontal disease, tooth and bone loss, and hard and soft tissue lesions in the oral cavity (1, 3). The most common effects reported by the World Dental Federation (FDI), were mouth and throat irritation and periodontal damage (11). Survey data from the US showed that e-cigarette use is prevalent among the young age-groups particularly those in middle and high school (12). Other countries in Europe, Asia and Africa have reported its prevalence and proposed legislation on its use and sale to persons under 18-years old (13). In the Caribbean region the available on the prevalence of e-cigarette use suggests an increase in younger age group (14, 15). According to survey data on youths in the Caribbean, Trinidad and Tobago had the highest prevalence of e-cigarette use in students (13–15 years) was 17.2% with the second and third highest being 11.7% and 11% in Jamaica and St. Lucia respectively. Other islands reported lower percentages (14). In Trinidad and Tobago efforts to control e-cigarette use especially among the young people included the Cancer Society's Anti-smoking/Anti-vaping campaign which sought to create awareness about the dangers of e-cigarette use and smoking (16). The aim of this research was to assess the prevalence of e-cigarette use and the attitudes toward and knowledge of its oral health effects among young adults in Trinidad and Tobago and implications for oral health promotion.

## Materials and methods

### Study design and sample selection

A cross-sectional survey was conducted among a convenience sample of undergraduate students of the. Faculty of Medical Sciences (FMS) at the University of the West Indies (UWI) in Trinidad and Tobago enrolled in Medicine, Dentistry, Veterinary Studies, Pharmacy, Optometry and Nursing during the academic year 2023–2024. Based on a student population for the 2023/2024 academic year of 1300 students, a confidence interval and 5% margin of error, the estimated sample size was 295 participants. Inclusion criteria were students enrolled in The UWI, Faculty of Medical Sciences during the period August 2023 to August 2024. Students enrolled in other faculties at the university, and those who were less than 18-years-old were excluded.

### Data collection

Data were collected using a self-administered, anonymous online questionnaire, adapted from an instrument used previously among dental students in the USA (7). Before data collection was undertaken, this questionnaire was piloted among a sample of 20 health sciences students and found to have acceptable face validity.

A link for the questionnaire was disseminated via a social media platform to all students in the included programs, via class representatives. The questionnaire contained thirty-three questions items including e-cigarette use, knowledge and attitudes to vaping and its effects on health and oral health. Question responses employed 5-point Likert scales: “strongly agree” = 5, “agree” = 4, “neither or disagree” = 3, “disagree” = 2, and “strongly disagree” = 1. (These included items on Knowledge: “extremely familiar” = 5 to “not familiar” = 1, Attitudes: “extremely important” = 5 to “not important” = 1, and Oral health rating: “excellent” = 5 to “very poor” = 1).

### Data analysis

Data processing and analyses were conducted using IBM© SPSS© Statistics Version 27 for Macintosh, to provide descriptive statistics.

### Ethical considerations

Ethical approval was obtained from The UWI Campus Research Ethics Committee. Informed consent was gained from individual participants. Data collection protocols included confidentiality and data security measures.

You may insert up to 5 heading levels into your manuscript as can be seen in “Styles” tab of this template. These formatting styles are meant as a guide, as long as the heading levels are clear, Frontiers style will be applied during typesetting.

## Results

### Demographics and prevalence of e-cigarette use

**Figure 1:**
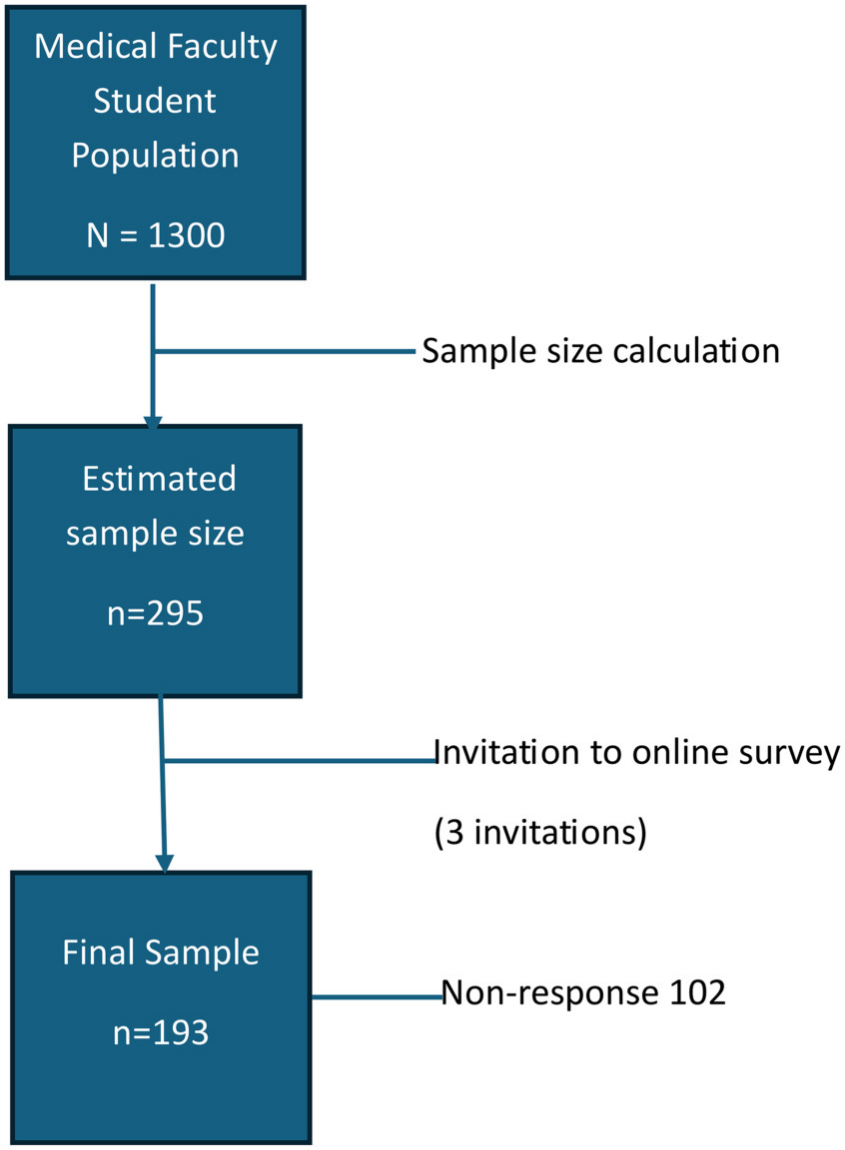
Sample size and response to the survey.

193 students from the estimated sample size of 295, participated in the survey. Following three rounds of survey link invitation reminders, this represented a 65% response rate (Figure 1).

The age range of participants was 19–25 years and mean age of 22 years. The majority of participants were female (75.1%) and most were from the programmes of medicine (42%), and dentistry (23.3%). Fifteen per cent of participants reported current use of e-cigarettes. The most popular device used were disposable pens (44.80%).

Participants were asked when they first began to vape and most reported “one year ago” (58.1%), followed by “more than five years ago” (29%). The three main reasons participants started vaping were “influence of friends”, “attractive flavours” and “stress” (65%–80%), followed by those who believed that it was less harmful than smoking (37.5%). The majority of those who reported vaping stated they acquired them from specialty stores (81%). Followed by friends (25%).

### Knowledge and attitudes to vaping and oral health

Most participants agreed with the statements: vaping may be addictive, delivers nicotine, delivers no chemicals and can affect your health (Table 1).

**Table 1:** Knowledge of e-cigarettes and health effects.

Statement	Frequency	Percent
Vaping is addictive		
Yes	176	91.20%
No	4	2.10%
Unsure	13	6.70%
Vaping delivers nicotine		
True	168	87.00%
False	6	3.10%
Unsure	19	9.80%
Vaping delivers no chemicals		
True	4	2.10%
False	169	87.60%
Unsure	20	10.40%
Vaping affects health		
True	189	97.90%
False	0	0.00%
Unsure	4	2.10%
Vaping is related to tooth decay		
True	99	51.30%
False	7	3.60%
Unsure	87	45.10%
Vaping is related to gum disease		
True	113	58.50%
False	7	3.60%
Unsure	73	37.80%

When asked about effects of vaping on oral health, over half of participants reported that vaping is related to tooth decay (51.3%) and gum disease (58.5%).

When asked to rate their familiarity with the chemicals contained in vaping devices (Table 2), the majority of respondents were slightly familiar (39.9%) while (25.4%) were moderately familiar and only (2.1%) were extremely familiar. Majority of participants stated that they were not at all familiar with research on vaping on oral health (44%) with some slightly familiar (33.2%) and the remainder being moderately or not at all familiar. In addition, (45.6%) were not at all familiar with the effect flavoured vaping products have on the health of their mouth (Table 2).

**Table 2:** Knowledge on contents of e-cigarettes.

Statement	Frequency	Percent
Familiarity with chemical contents.		
Not familiar	47	24.40%
Slightly familiar	77	39.90%
Moderately familiar	49	25.40%
Very familiar	16	8.30%
Extremely familiar	4	2.10%
Familiarity with research on oral health effects		
Not familiar	85	44.00%
Slightly familiar	64	33.20%
Moderately familiar	35	18.10%
Very familiar	8	4.10%
Extremely familiar	1	0.50%
Familiarity with effect of flavoured vaping products		
Not familiar	88	45.60%
Slightly familiar	56	29.00%
Moderately familiar	35	18.10%
Very familiar	10	5.20%
Extremely familiar	4	2.10%

### Associations between demographic and knowledge variables and current e-cigarette use

An independent sample *t*-test showed no significant difference in mean age between current users of e-cigarettes and non-users, *p* = 0.661. Chi-square analyses revealed a significant association between gender and current vaping status, with female students more likely to report having used e-cigarettes than male *p* = 0.003. There were no significant association found between academic programmes and vaping, *p* = 0.360. Participants with low familiarity on effects of vaping on oral health were more likely to use e-cigarettes *p* = 0.030.

### Advice and education on e-cigarettes and oral health

The majority of participants stated that the value of oral health, learning about the connection between vaping and oral health and the importance of speaking to a dentist about vaping on oral health is extremely important (66.80%, 37.30% and 37.80% respectively) (Table 3).

**Table 3:** Impact on oral health of e-cigarettes.

Statement	Frequency	Percent
Value of personal oral health		
Not important	2	1.00%
Slightly important	1	0.50%
Moderately important	9	4.70%
Very important	52	26.90%
Extremely important	129	66.80%
Importance of Education on vaping and oral health		
Not important	6	3.10%
Slightly important	14	7.30%
Moderately important	36	18.70%
Very important	65	33.70%
Extremely important	72	37.30%
Importance of speaking with a dentist on effect of vaping on oral health		
Not important	9	4.70%
Slightly important	16	8.30%
Moderately important	33	17.10%
Very important	62	32.10%
Extremely important	73	37.80%

However, 93.2% of those who reported vaping had never consulted their dentist about the effects of vaping on oral health.

Most participants stated that they visit the dentist at least once per year (31.1%), followed by every 2–5 years (26.9%) with only 10% stating they never visit the dentist.

The most recent dental visits were for a routine examination (59.1%). Eight five per cent brushed their teeth at least 2 times per day.

Most participants rated their oral health as good (52.8%), followed by fair (30.6%) and excellent (13%) (Table 4). Over a third of participants believed that vaping has a moderate effect on oral health (34.70%) (Table 4) and would be willing to speak to an oral health professional on its negative impact on the health of the mouth (44%). Also, participants stated that they would be willing to cut down (51.30%) if they knew that vaping was harmful to one's oral health (Table 4).

**Table 4:** Vaping and self-rated oral health.

Statement	Frequency	Percent
Willingness to cut down on vaping if believed harmful to oral health		
Strongly disagree	7	3.60%
Disagree	4	2.10%
Neither agree nor disagree	33	17.10%
Agree	50	25.90%
Strongly agree	99	51.30%
Belief that vaping impacts the health of your mouth		
Minor effect	13	6.70%
Slight effect	17	8.80%
Moderate effect	67	34.70%
Strong effect	60	31.10%
Major effect	36	18.70%
Overall health of your mouth		
Very poor	2	1.00%
Poor	5	2.60%
Fair	59	30.00%
Good	102	52.80%
Excellent	25	13.00%

The majority of participants stated they would be happy to receive information on the health effects of vaping mainly through oral health professionals (85%), followed by a healthcare provider (71%) or through internet/social media (69.40%).

## Discussion

The prevalence of e-cigarette use in this sample of university students was similar to data from school age students in the Caribbean (14), lower than previous data for young adults in Trinidad (1).and internationally, lower than university students in Malaysia (17) Ireland (18), China (19), US (20) Palestine (21) but higher than New Zealand (22).

Among participants who reported using e-cigarettes, they attributed their use due to ease of availability among other factors. Disposable pens were seen as the most popular type of e-cigarette, which is similar to the literature (13). This indicates that those who vaped prefer convenient and easily accessible means of delivery. Among this sample the main reasons for starting to vape were stress, influence from friends, attractive flavours and a belief it was healthier than smoking. Although research has shown vaping most often starts in high school participants in this study who used e-cigarettes, stated that they began using vapes one year ago. This may indicate starting vaping around the early phase of university life, possibly due to additional stress and increased workload.

The majority of participants agreed that vaping may be addictive, delivers nicotine or chemicals and can affect health. This suggests these health science students were generally aware of the possible risks of e-cigarette use.

Inferential statistics revealed significant gender differences in e-cigarette use, with more females having reported current use. This contrasts with some global and regional studies that report higher prevalence among males (12, 14). It is important to note that participants were predominantly female, therefore the observed gender association may reflect the sample rather than a true behavioral trend.

Regarding oral health, most participants agreed that it was important and reported having good oral health and oral hygiene habits, including regularly visiting the dentist. With respect to vaping effects on oral health, the responses were not as clear, showing general unfamiliarity on the issue. The majority of participants would cut down or quit vaping if it is known to affect oral health. Similar to research in the US (8), speaking to a dental professional about risks of vaping for oral health was seen as very important by most participants which indicates an interest in acquiring more information on the issue. This also supports the view that with the increasing popularity of e-cigarettes, the importance of oral health promotion and advice in the clinical setting has become more critical (23).

Improving knowledge among health science students on these issues could be addressed incorporating training on smoking cessation and vaping in their undergraduate progammes. These educational interventions could include seminars, online interactive modules, case-based learning and practical assessments such as objectives structured clinical exams (OSCE).

### Limitations

The use of a convenience sample from a single institution and response rate may limit the generalizability of the findings of this research to other Caribbean populations or health sciences students globally. Including health sciences training institutions from across the region in a large sample frame and employing random sampling, could provide further insights.

## Conclusion

Among this sample of health sciences students, e-cigarette use was low, however there was limited knowledge of its effects on oral health. Adverse effects of e-cigarette use on oral health should be included in health promotion initiatives aimed at reducing or preventing vaping among younger adults.

## Data Availability

The datasets presented in this article are not readily available because the data is part of a student research project. Further questions can be directed to the corresponding author.

## References

[1] Abbott AJ Reibel YG Arnett MC Marka N Drake MA. Oral and systemic health implications of electronic cigarette usage as compared to conventional tobacco cigarettes: a review of the literature. J Dent Hygiene. (2023) 97:21–35. Available at: https://jdh.adha.org/content/97/4/21 37553278

[2] World Health Organization. Tobacco: E-cigarettes (2022). Available at: https://www.who.int/news-room/questions-and-answers/item/tobacco-e-cigarettes (Accessed October 1, 2024).

[3] Amaral AL Lwaleed BA Andrade SA. Electronic nicotine delivery systems (ENDS): a strategy for smoking cessation or a new risk factor for oral health? Evid Based Dent. (2023) 24(4):188–9. 10.1038/s41432-023-00929-w 37674038

[4] Amaral AL Lwaleed BA Andrade SA. Is there evidence that e-cigarettes promote an increased risk of dental caries? Evid Based Dent. (2023) 24(4):170–1. 10.1038/s41432-023-00933-0 37704804

[5] Grana R Benowitz N Glantz SA. E-cigarettes: a scientific review. Circulation. (2014) 129:1972–86. 10.1161/CIRCULATIONAHA.114.007667 24821826 PMC4018182

[6] Hua M Alfi M Talbot P. Health-related effects reported by electronic cigarette users in online forums. J Med Internet Res. (2013) 15:e59. 10.2196/jmir.2324 23567935 PMC3636314

[7] Amaral AL da Costa Andrade PA Lwaleed BA Andrade SA. Impacts of smoking on oral health-what is the role of the dental team in smoking cessation? Evid Based Dent. (2023) 24(4):186–7. 10.1038/s41432-023-00930-3 37679450

[8] Martell KM Boyd LD Giblin-Scanlon LJ Vineyard J. Knowledge, attitudes, and practices of young adults regarding the impact of electronic cigarette use on oral health. J Am Dent Assoc. (2020) 151:903–11. 10.1016/j.adaj.2020.08.002 33228883

[9] Al-abdouh A Phillips E Allison MG. E-cigarette or vaping product use-associated lung injury: a severe case that responded to corticosteroid treatment. Cureus. (2020) 12:e11544. 10.7759/cureus.11544 33365213 PMC7748588

[10] Belok SH Parikh R Bernardo J Kathuria H. E-cigarette, or vaping, product use-associated lung injury: a review. Pneumonia. (2020) 12:12. 10.1186/s41479-020-00075-2 33110741 PMC7585559

[11] FDI World Dental Federation. The effects of e-cigarettes on oral health (2021). Available at: http://www.fdiworlddental.org/sites/default/files/2021-07/The%20Effects%20of%20E-cigarettes%20on%20Oral%20Health%20-%20Fact%20Sheet.pdf (Accessed October 25, 2023).

[12] FDA US food and Drug Administration. Results from the Annual National Youth Tobacco Survey (2022). Available at: https://www.fda.gov/tobacco-products/youth-and-tobacco/results-annual-national-youth-tobacco-survey (Accessed October 26, 2023).

[13] Graham-DeMello A Hoek J Drew J. How do underage youth access e-cigarettes in settings with minimum age sales restriction laws? A scoping review. BMC Public Health. (2023) 23:1809. 10.1186/s12889-023-16755-9 37723457 PMC10506222

[14] Healthy Caribbean Coalition. Tobacco use among Caribbean youths (2021). Available at: https://www.healthycaribbean.org/tobacco-use-among-caribbean-youths/ (Accessed October 26, 2023).

[15] Dhandoolal R De Gannes S Dhanoolal A Desaine M Dukhoo D Duncombe S Electronic cigarette use among emerging and young west Indian adults. EMJ Respir. (2017) 5:108–15. 10.33590/emjrespir/10310313

[16] Trinidad and Tobago Cancer Society. Take a breath initiative – Trinidad & Tobago Cancer Society (2024). Available at: https://cancertt.com/take-a-breath/ (Accessed October 1, 2024).

[17] Puteh SEW Manap RA Hassan TM Ahmad IS Idris I Sham FM The use of e-cigarettes among university students in Malaysia. Tob Induc Dis. (2018) 16:57. 10.18332/tid/99539 31516454 PMC6659562

[18] Hayes CB Kennedy N Poon D Frain J Lee A Lee C Factors associated with current e-cigarette use in an Irish university and attitudes to proposed legislative change. Tob Prev Cessat. (2023) 9(Supplement 2):A83. 10.18332/tpc/172735

[19] Song H Yang X Yang W Dai Y Duan K Jiang X Cigarettes smoking and e-cigarettes using among university students: a cross-section survey in Guangzhou, China, 2021. BMC Public Health. (2023) 23:438. 10.1186/s12889-023-15350-2 36882716 PMC9990220

[20] Vilcassim MJR Stowe S Zierold KM. Perception of health risks of electronic cigarette use among college students: examining the roles of sex, field of study, vaping device type, and their associations. J Community Health. (2025) 50:23–30. 10.1007/s10900-024-01393-y 39179760 PMC11805785

[21] Ghanim M Rabayaa M Abuawad M Saeedi M Amer J. E-cigarette use among university students in Palestine: prevalence, knowledge and determinant factors. PLoS One. (2024) 19:5. 10.1371/journal.pone.0302946 PMC11078419 38718008

[22] Wamamili B Wallace-Bell M Richardson A Grace RC Coope P Electronic cigarette use among university students aged 18–24 years in New Zealand: results of a 2018 national cross-sectional survey. BMJ Open. (2020) 10:e035093. 10.1136/bmjopen-2019-035093 32571858 PMC7311043

[23] Cameron A Yip HM Garg M. Current thinking about the effects of e-cigarettes on oral cancer risk. BDJ Team. (2024) 236:397–400. 10.1038/s41415-024-7124-2 38459320

